# Supplementary material for: Patterns of Intron Gain and Loss in Fungi
Source: PLoS Biol. 2004 Nov 30;2(12):e422. doi: 10.1371/journal.pbio.0020422 (PMC532390; doi:10.1371/journal.pbio.0020422)
Supplement: Table S1 — Also available at http://genes.mit.edu/NielsenEtAl/. (4.3 MB ZIP). [file pbio.0020422.st001.zip › NielsenEtAl/html/429.html]

AN4780.1.NCU00590.1.MG10319.1.FG08689.1


```
 CLUSTAL W (1.82) Multiple Sequence Alignments - Introns Inserted


Sequence 1: NCU00590.1	262 aa
Sequence 2: FG08689.1	229 aa
Sequence 3: MG10319.1	159 aa
Sequence 4: AN4780.1	241 aa
Alignment Length: 267 aa
Number Identitical Residues: 69 aa
Alignment Score (without introns) 4016


MG10319.1 	---------------------------------------~--------------------
NCU00590.1	--MNQQIPLNYEASAGNTHMRITSELPPEVVQCLENARF~LHLATCVDNMPHVSLMNYTY
FG08689.1 	---MEQIPLNSEASVGDTHKQTTSTLPRDVVQCLDNARF0LHLATCTDNMPHVSLMNYTY
AN4780.1  	MSDSLNPPLSYEASATTTHRHVATTLPPEVSSCLKNSRF0LHLATCDGLTPHISLMSYTY
          	 :   . . . .::   :    ::  . .  .. ..:      ::. .  .  :  . : 

MG10319.1 	-------------MTTNAASKKTNNLIINPNVSLLVHDW1VSHRPPS-SNRRPSG--GSP
NCU00590.1	LPSSPYSDSPIIVMTTNPASKKMNNLVANPNVSLLVHDW1VSHRPSTSHQRRPSS--GSP
FG08689.1 	LPSSPYSNYPVIIMTTNPASKKTSNLVTNPNVSLLVHDW1VSHRPPT-HGRRPSG--GSP
AN4780.1  	LPSTPFDPYPTIIMTTNSSSRKTTHLQTNPRVSLLVHDW~VSHRPPTRASNPGNTRDGSP
          	 .::. .  .   ****.:*:* .:*  **.******** *****.:   .  .  .***

MG10319.1 	GP-EHR-SSLASLLLNLNTSSISSISATINGSARLADAGSEEESFFKQQHLDNNTFED--
NCU00590.1	GPSEHRSSSLAALLFNLNTSAVSSISATINGSARLVDRGSEEERFYREKHLENNTFDSDP
FG08689.1 	GP-EHR-SSLASLLLNLNTTALSSISATIGGAARLVPSGTEEEKYYREQHLENNTFD---
AN4780.1  	PPAATR-SSLASLLLNLNTSALSSISTTITGEARFLQPNSEEETWCKERHLENNTFEE--
          	 *:  * ****:**:****:::****:** * **:   .:*** : :::**:****:.  

MG10319.1 	--SSAGASIFNS---------------TPADG--ARPTFVEGEDVRVVVVKIQDVRISDW
NCU00590.1	QPSSILSSIRRGSTAGQPTSMSRGGQGAGEEGDYGRERYVADEEVRVVVVGIRDVRIADW
FG08689.1 	--ESTTESFQRE-------------NGLVEDG--GRSCFVAGEEVRVVSVDIKDVRISDW
AN4780.1  	----EMSTFGQA----------------QQPGQ-RRPSLSIDDDVRVITIRVREGRLADW
          	       :: .                    *.  *     .::***: : ::: *::**

MG10319.1 	KGSVRDYVIVP-----SEP---MVNGV--
NCU00590.1	KGTVQDWVIAPPEGRSEGTNETLVNGIR-
FG08689.1 	KGTVRDWEIVP-----EGS---MVNGT--
AN4780.1  	KGGVRDWLVVQE--GEEGPDETTPNGVVL
          	** *:*: :.     .. ...:  **
```
